# Impact of Hormonal Contraceptives on Cervical T-helper 17 Phenotype and Function in Adolescents: Results from a Randomized, Crossover Study Comparing Long-acting Injectable Norethisterone Oenanthate (NET-EN), Combined Oral Contraceptive Pills, and Combined Contraceptive Vaginal Rings

**DOI:** 10.1093/cid/ciz1063

**Published:** 2019-11-02

**Authors:** Iyaloo N Konstantinus, Christina Balle, Shameem Z Jaumdally, Hoyam Galmieldien, Tanya Pidwell, Lindi Masson, Ramla F Tanko, Anna-Ursula Happel, Musalula Sinkala, Landon Myer, Steven E Bosinger, Katherine Gill, Linda-Gail Bekker, Heather B Jaspan, Jo-Ann S Passmore

**Affiliations:** 1 Institute of Infectious Diseases and Molecular Medicine, University of Cape Town, Cape Town, South Africa; 2 Desmond Tutu Human Immunodeficiency Virus Centre, University of Cape Town, Cape Town, South Africa; 3 Division of Epidemiology and Biostatistics, School of Public Health and Family Medicine, University of Cape Town, Cape Town, South Africa; 4 Department of Pathology & Laboratory Medicine, Emory University School of Medicine, Atlanta, USA; 5 Division of Microbiology and Immunology, Yerkes National Primate Research Center, Atlanta, USA; 6 Seattle Children’s Research Institute, Seattle, Washington, USA; 7 University of Washington Department of Pediatrics and Global Health, Seattle, Washington, USA; 8 National Health Laboratory Service, Cape Town, South Africa

**Keywords:** Th17, NET-EN, CCVR, FSH, cytokines

## Abstract

**Background:**

Adolescents in sub-Saharan Africa are at risk for human immunodeficiency virus (HIV) infection and unintended pregnancies. Observational studies suggest that injectable hormonal contraceptives (HCs) increase the HIV risk, although their effects on genital inflammation, particularly HIV-susceptible T-helper 17 (Th17) cells, are unknown. In a randomized crossover study, the effect of injectable norethisterone oenanthate (NET-EN), combined contraceptive vaginal rings (CCVR; NuvaRing), and combined oral contraceptive pills (COCPs) on cervical Th17 cells and cytokines were compared.

**Methods:**

Adolescents (n = 130; 15–19 years) were randomly assigned 1:1:1 to NET-EN, CCVR, or COCPs for 16 weeks, then subsequently crossed over to another HC for 16 weeks. Estrogen, follicular stimulating hormone (FSH), and luteinizing hormone (LH) levels were measured. Chemokine receptor 5 (CCR5), human leukocyte antigen (HLA) DR isotope, and cluster of differentiation 38 (CD38) expression by cervical cytobrush-derived CD4+ T cells was assessed by fluorescence-activated cell sorting. Th17 cells were defined as CCR6+ and CCR10-. Cervicovaginal Th17-related cytokines were measured by Luminex.

**Results:**

CCVR use for the first 16 weeks was associated with reduced Th17 frequencies and lower FSH and LH concentrations, as compared to NET-EN and COCPs, with FSH concentrations and Th17 frequencies correlating significantly. However, Th17-related cytokine concentrations (interleukin [IL]-21, IL-1β, tumor necrosis factor–α, interferon-γ) and CCR5, HLA-DR, CD38, and Th17 frequencies were significantly higher in CCVR than NET-EN and COCP. At crossover, CCVR users changing to COCPs or NET-EN did not resolve activation or cytokines, although switching from COCP to CCVRs increased cytokine concentrations.

**Conclusions:**

CCVR use altered endogenous hormone levels and associated cervical Th17 cell frequencies to a greater extent than use of NET-EN or COCPs, although Th17 cells were more activated and Th17-related cytokine concentrations were elevated. While CCVRs may impact the HIV risk by regulating Th17 numbers, increased activation and inflammation may balance any risk gains.

## Teaser

Adolescents using injectable norethisterone oenanthate had similar endogenous hormones, genital cytokines, and Th17 cell frequencies as combined oral contraceptive pill users. NuvaRing use reduced endogenous hormones and lowered cervical frequencies of Th17 cells but enhanced Th17 activation and Th17-related cytokines.

Young and adolescent females in sub-Saharan Africa are at higher risk for human immunodeficiency virus (HIV) than young males [1]. Adolescence is also a time of sexual and hormonal contraceptives (HCs) debut. Although HCs are critical for preventing unintended pregnancies and maternal/neonatal mortality, observational studies have reported an increased HIV risk associated with certain formulations, such as progestin-only injectable depot medroxyprogesterone acetate (DMPA) [2, 3]. Although the Evidence for Contraceptive Options and HIV Outcomes randomized, open-label trial found no significant difference in HIV risks in women using DMPA, copper intrauterine devices (cIUDs), or levonorgestrel implants, the HIV incidence was highest in those using DMPA (4.2; 95% confidence interval [CI], 3.5–4.9), as compared to cIUDs (3.9; 95% CI, 3.3–4.7; hazard ratio [HR], 1.04; 95% CI, .82–1.33; *P* = .72) or levonorgestrel (3.3; 95% CI, 2.7–4.0; HR, 1.23; 95% CI, 1.0–1.6; *P* = .097) [4]. The cIUDs were included as the non-hormonal control rather than a placebo, although cIUDs may cause inflammation [5], which may have influenced the HIV risk. Considering this, we propose that a knowledge gap remains about the biological mechanisms of HIV risk associated with HCs (specifically the related progestin injectable norethisterone oenanthate [NET-EN]), particularly in adolescents naive to contraceptives. In vitro and clinical studies have produced conflicting results as to whether HCs influence HIV target cells. DMPA has been associated with elevated cervical CD4+ T-cell frequencies and expression of the HIV co-receptor chemokine receptor 5 (CCR5) on these cells [6–9], although data on other forms of HCs and in adolescents remain limited.

Genital inflammation has been associated with an HIV risk in women, with CCR5-binding chemokines (macrophage inflammatory protein [MIP]-1α and MIP-1β) being the strongest predictors [10]. These chemokines have been linked with increased frequencies of activated cervical CD4+ T cells [11]. Genital tract CD4+ T-cell subsets may not be equally susceptible to HIV infection [12, 13], with T-helper 17 (Th17) cells preferentially infected and rapidly depleted during HIV infection [14–16]. Th17 cells selectively express CCR6, CCR4, CD161, and interleukin (IL)-23R, as well as the transcription factor receptor tyrosine kinase-like orphan receptors (ROR)-γt, which are involved in Th17 differentiation [16–18]. The homing receptor CCR6, involved in Th17 recruitment to inflammatory sites, is among the best-established phenotypic markers for human Th17 cells. Th17 cells contribute to the first line of defense against pathogens at the mucosal surfaces, with protection mediated by IL-17A, IL-17F, IL-21, and IL-22 [19]. Although Th17 cells provide protection from pathogens, their presence in the genital tract might influence the HIV risk.

We hypothesized that high-dose, progestin-only NET-EN would effect genital CD4+ target cell activation and cytokines to a greater extent than other HCs, which may influence HIV risk in adolescents. We focused on Th17 cells because they are important at the mucosa and are susceptible to HIV [16]. We compared the influence of NET-EN to estrogen/progestin-combination contraceptives, including combined oral contraceptive pills (COCPs) and combined contraceptive vaginal rings (CCVR), on cervical Th17 cell frequencies and activation in a randomized crossover study in adolescents.

## MATERIALS AND METHODS

Between 2015–2017, adolescent women living without HIV (15–19 years) were recruited to investigate the safety and acceptability of a CCVR (NuvaRing; Merck), a vaginal ring inserted monthly, compared to bi-monthly, injectable NET-EN and COCPs, Triphasil, or Nordette (clinicaltrials.gov/NCT02404038). The Human Research Committee of the University of Cape Town approved the study (UCT HREC 801/2014), and adolescents aged 18–19 provided informed, written consent, while those <18 years provided written assent and consent was obtained from parents/guardians. The inclusion criteria were living without HIV, being sexually active, not being pregnant, having no symptomatic sexually transmitted infection (STI) in the previous 40 days (self-reported), and not using non-study products/objects vaginally during study. At enrollment, each participant completed a nurse-administered questionnaire. Adolescents came in for a screening visit (baseline) and, if eligible, were randomly assigned (1:1:1) to receive NET-EN (200 mg), CCVR (Nuvaring; etonogestrel/ethinyl estradiol), or COCPs (Triphasil or Nordette; both ethinyl estradiol and levonorgestrel) for 16 weeks. Those receiving NET-EN or COCPs then switched to CCVR, while those receiving CCVR chose between NET-EN or COCPs for the remaining 16 weeks. There was no HC washout prior to or during the study, to avoid unintended pregnancies. At each visit, a menstrual cup (Softcup, Evofem Inc) was collected for cervicovaginal cytokine measurements, a cervical cytobrush was collected for mucosal T-cell isolation, a vulvovaginal swab was collected for STI screening, and blood was collected for the measurement of HSV-2 antibodies and endogenous hormones. At baseline and exit visit, estrogen (E2), follicular stimulating hormone (FSH), and luteinizing hormone (LH).

### Screening for Endogenous Hormones, Sexually Transmitted Infections, and Bacterial Vaginosis

Endogenous hormones (E2, LH, FSH) were measured in plasma by electrochemiluminescence (Cobas; National Health Laboratory Service, Groote Schuur Hospital, Cape Town). Vulvovaginal swabs were screened for *Chlamydia trachomatis*, *Neisseria gonorrhoeae*, *Mycoplasma genitalium*, and *Trichomonas vaginalis* by multiplex polymerase chain reaction [11], and screenings were performed at the same study visits as mucosal sample collections. Bacterial vaginosis (BV) was determined by Nugent scoring, with 7–10 being considered BV positive (BV+). Fungal hyphae on Gram stains suggested vaginal yeast infections. Vaginal pH was measured using color-fixed indicator strips (Macherey-Nagel, Düren, Germany). The STI/BV+ adolescents were treated according to national guidelines.

### Measurement of Cytokines

Cervicovaginal cytokine concentrations were measured using Luminex (Bio-Plex Pro Human Th17 cytokine panel, Bio-Rad Laboratories, Hercules, CA), which included cytokines associated with Th17 cell differentiation (IL-1β, IL-6, tumor necrosis factor [TNF]-α, IL-23, and IL-33), those produced by Th17 cells (IL-17A, IL-17F, IL-21, and IL-22), and those associated with Th17 cell regulation (IL-25, IL-31, interferon [IFN]-γ, and soluble CD40 ligand). Data were collected using a Bio-plex Suspension Array reader, as previously described [11].

### Flow Cytometry

Cervical cells were obtained from Digene cytobrushes, processed within 4 hours, and stained immediately [11]. The antibodies included: APC-H7-CD3, BV605-CCR6, and APC-CCR5 (all from BD Biosciences, San Jose, CA); BV711-CD8, PE-CCR10, and Alexa-700-human leukocyte antigen (HLA) DR isotope (Biolegend, San Diego, CA); PE-Cy5.5-CD4 (Invitrogen, Carlsbad, CA); and PE-Cy7-cluster of differentiation 38 (CD38) (eBioscience, San Diego, CA). Staining for LIVE-DEAD cells, Pac-blue-CD14, and Pac-blue-CD19 was performed to exclude dead cells, monocytes, and B-cells, respectively (all from Invitrogen). Cells were acquired using the FORTESSA (BD Immunocytometry Systems, San Jose, CA). FlowJo v9.9.3 (FlowJo, LLC, Ashland, OR) was used for the data analysis. The gating strategy is shown in Supplementary Figure 1.

### Statistical Analyses

GraphPad Prism6 (GraphPad software), STATA version 12.0 (StataCorp), and MatLab were used for analyses. Mann-Whitney U, Spearman rank test, and Wilcoxon signed-rank tests were used for paired and unpaired analyses, respectively. *P* values < .05 were considered significant, and a multiple comparisons adjustment was done using the Benjamini-Hochberg procedure.

## RESULTS

We randomly assigned 130 adolescents (median, 17 years; interquartile range, 16–18 years) into 3 HC arms: NET-EN (45/130), CCVR (45/130), and COCPs (40/130). Of these, 107 completed the crossover visit (82.3%; 16 weeks) and 92 completed the study (70.8%; 32 weeks). No significant differences in demographic, sexual, or clinical characteristics between HC groups were noted at enrollment (Table 1).

**Table 1.** Baseline Characteristics of Adolescents, According to Randomization Arm

	All	NET-EN, n = 45	COCPs, n = 40	CCVR, n = 45
Age, median (IQR)	17 (16–18)	17 (16–18)	17 (16–18)	17 (16–18)
BMI, kg/m^2^, median (IQR)	25.2 (21.9–28.4)	25.0 (22.5–29.1)	25.7 (21.5–28.3)	25.0 (21.9–27.8)
Vaginal pH, mean (SD)	4.8 (4.5–5.2)	5.0 (4.3–5.6)	4.8 (4.3–5.4)	4.8 (4.4–5.2)
Genital infections, n (%)				
Any STIs	56 (43.1%)	22 (48.9%)	15 (37.5%)	19 (42.2%)
*Chlamydia trachomatis*	43 (33.1%)	17 (37.7%)	12 (30.0%)	14 (31.1%)
*Neisseria gonorrhoeae*	13 (10.0%)	5 (11.1%)	3 (7.5%)	5 (11.1%)
*Trichomonas vaginalis*	13 (10.0%)	5 (11.1%)	4 (10.0%)	4 (8.8%)
*Mycoplasma genitalium*	3 (2.3%)	2 (4.4%)	1 (2.5%)	0 (0%)
HSV-2 IgG	39 (30.0%)	13 (28.8%)	14 (35.0%)	12 (26.7%)
Presence of yeast hyphae	20 (15.4%)	4 (8.8%)	6 (15.0%)	10 (22.2%)
BV status, n (%)				
BV-, Nugent 0–3	61 (46.9%)	18 (40.0%)	20 (48.8%)	23 (51.1%)
Intermediate, Nugent 4–6	14 (10.8%)	7 (15.6%)	4 (9.8%)	3 (6.6%)
BV+, Nugent 7–10	55 (42.3%)	20 (44.4%)	16 (40.0%)	19 (43.2%)
Prior HC method, n (%)				
Never	5 (3.8%)	2 (4.4%)	1 (2.5%)	2 (4.4%)
Not currently	26 (20.0%)	10 (22.2%)	10 (25.0%)	6 (13.3%)
DMPA	19 (14.6%)	7 (15.9%)	7 (17.5%)	5 (11.1%)
NET-EN	69 (53.1%)	21 (46.7%)	20 (50.0%)	28 (62.2%)
COCPs	6 (4.6%)	2 (4.5%)	1 (2.5%)	3 (6.7%)
Implanon	3 (2.3%)	2 (4.5%)	0 (0%)	1 (2.2%)
Endogenous hormone concentrations				
Luteinizing hormone, IU/L	…	4.4 (2.8–5.6)	4.7 (2.9–5.7)	4.7 (2.9–5.7)
FSH, IU/L	…	4.0 (1.9–6.6)	4.4 (2.0–5.9)	4.4 (2.0–5.9)
Estrogen, pmol/L	…	107 (85–157)	104 (74–155)	104 (74–155)
Sexual risk behavior, n (%)				
Age of sexual debut, median (IQR)	15 (14–16)	15 (14–16)	15 (14–16)	15 (14–16)
Condom use with last PV intercourse	78 (60.0%)	26 (60.5%)	25 (62.5%)	27 (60.0%)
Multiple sexual partners	1 (.7%)	1 (2.2%)	0 (0%)	0 (0%)
Vaginal practices, n (%)				
Use of any products	7 (5.4%)	4 (8.8%)	2 (2.5%)	1 (4.6%)
Washing with soap	12 (9.2%)	6 (13.3%)	2 (5.1%)	4 (8.8%)
Tampon use	8 (6.2%)	5 (11.1%)	1 (2.5%)	2 (4.6%)
Washing with water	16 (12.3%)	6 (13.3%)	5 (12.8%)	5 (11.1%)
Douching	1 (.7%)	1 (2.2%)	0 (0%)	0 (0%)

Abbreviations: -, negative; +, positive; BMI, body mass index; BV, bacterial vaginosis; CCVR, combined contraceptive vaginal ring; COCPs, combined oral contraceptive pills; DMPA, depot medroxyprogesterone acetate; FSH, follicular stimulating hormone; HC, hormonal contraceptive; HSV-2, herpes simplex virus type-2; IgG, immunoglobin G; IQR, interquartile range; NET-EN, injectable norethisterone oenanthate; PV, penile-vaginal sex; SD, standard deviation; STIs, sexually transmitted infections.

### T-helper 17 Cells and T-helper 17–Related Cytokines in the Genital Tracts of Adolescents at Baseline

**Figure 1.**
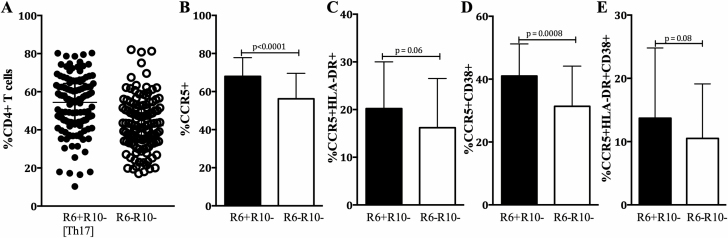
Cervical Th17 cells were identified by expression of CCR6 and CCR10 (CCR6+ CCR10-). *A*, Cervical CD4, CD3, and T-cell frequencies were defined as CCR6+ CCR10- (Th17) or CCR6- CCR10- (enriched for Th1/2 cells). The expression of CCR5 is shown (*B*) alone and in combination with (*C*) HLA-DR, (*D*) CD38, or (*E*) HLA-DR and CD38 by Th17 cells (CCR6+ CCR10-; black bars) and Th1/2-like T cells (CCR6- CCR10-; clear bars). Mann-Whitney U tests were applied to compare the markers between the groups, and *P* values ≤ .05 were considered significant. Abbreviation: Th17, T-helper 17.

At baseline, the majority of CD3+ CD4+ cervical cytobrush cells were CCR6+ CCR10- Th17-like cells (54.4%), followed by 42.2% that were CCR6- CCR10- T cells (enriched for Th1 cells; Figure 1A). A minority were CCR10+ (1.2% CCR6+ CCR10+; 0.8% CCR6- CCR10+; data not shown). Higher frequencies of total and highly activated (CD38+) cervical Th17 cells expressed CCR5, compared to CCR6- CCR10- CD4+ T cells (*P* < .0001; Figure 1B; *P* = .0008; Figure 1D), while no differences were observed between those co-expressing CCR5/HLA-DR or CCR5/HLA-DR/CD38 (Figures 1C and E). Frequencies of cervical Th17 cells, activation, and CCR5 expression were similar across HC arms at enrollment (Supplementary Table 1).

While endogenous hormone levels were similar across arms at enrollment (Table 1), plasma FSH concentrations were positively associated with Th17 cell frequencies (Spearman Rho = 0.748; *P* = .030; Supplementary Table 2), although E2 and LH were not. The activation of Th17 (CD38+ HLA-DR+) was weakly, negatively associated with endogenous hormone concentrations (E2 Rho = −0.229; *P* = .013; LH Rho = −0.208, *P* = .025; Supplementary Table 2).

The best-described effector functions of Th17 cells include the production of cytokines, including IL-17A, IL-17F, IL-21, and IL-22 [18]. The baseline concentrations of Th17-related cytokines were similar across arms (Supplementary Table 3).

### Comparison of T-helper 17 Frequencies and Activation by Study Arm

**Figure 2.**
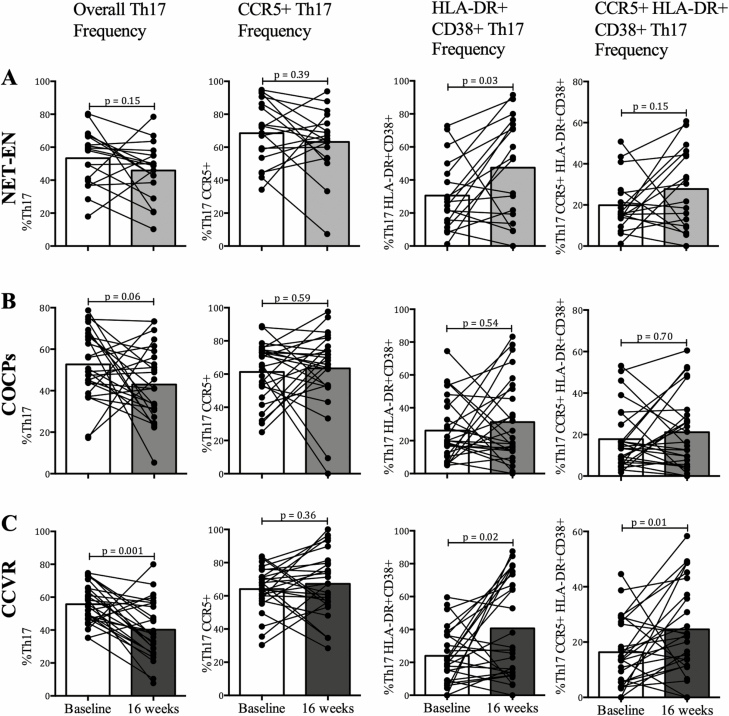
Phenotype of Th17 cells before (baseline) and after (16 weeks) being on (*A*) NET-EN, (*B*) COCPs, and (*C*) CCVR. The frequencies of Th17 cells and expression of CCR5, CD38, and HLA-DR on these cells were assessed before and after 16 weeks of adolescents being on the randomized HC. A Wilcoxon matched pairs signed rank test was applied and a *P* value ≤ .05 was considered significant. Abbreviations: CCVR, combined contraceptive vaginal ring; COCPs, combined oral contraceptive pills; HC, hormonal contraceptive; NET-EN, injectable norethisterone oenanthate; Th17, T-helper 17.

In an Intention to treat analysis at crossover (16 weeks), adolescents assigned to NET-EN and COCPs had similar cervical Th17 frequencies and CCR5 expression, which did not differ from baseline (Figures 2A and B). However, those randomized to NET-EN had higher frequencies of activated HLA-DR/CD38 Th17 cells, compared to their baselines, after 16 weeks (*P* = .03; Figure 2A), although the differences were not significant in the total CD4+ T cells (Supplementary Figure 2*A*). In contrast, adolescents assigned to CCVR had significantly lower frequencies of Th17 cells after 16 weeks, compared to baseline (*P* = .001), although they also had a significant increase in highly activated cervical Th17 cell frequencies (HLA-DR/CD38; *P* = .02), including those expressing the CCR5 (*P* = .01; Figure 2C). This change was also evident in the total CD4 population (all CD4+ T cells, *P* = .03 and all highly-activated CD4+ T cells [CD38+HLA-DR+] *P* = .03; Supplementary Figure 2*C*). Although we have reported that STIs/BV influence genital cytokines [10], their prevalences at baseline were similarly distributed across arms (Table 1) and did not differ at 16 weeks (Supplementary Table 4).

Adolescents assigned to NET-EN had similar concentrations of endogenous hormones as COCP users (Supplementary Table 4). Adolescents using CCVRs, in contrast, had significantly lower concentrations of LH (*P* < .0001) and FSH (*P* < .0001) than those using either COCP or NET-EN, with less pronounced differences for E2 (*P* = .05). Lower frequencies of Th17 cells in CCVR users may have been influenced by lower FSH concentrations, since (1) FSH was positively associated with cervical Th17 frequencies (Supplementary Table 2); and (2) adolescents using CCVRs had lower FSH concentrations (Supplementary Table 4). Since LH and E2 dampened Th17 activation (significantly so for LH; Supplementary Table 2), the lower LH and E2 concentrations associated with CCVR use may have contributed to their increased activation.

### Impact of Hormonal Contraceptives Arm on T-helper 17–Related Cytokines

**Figure 3.**
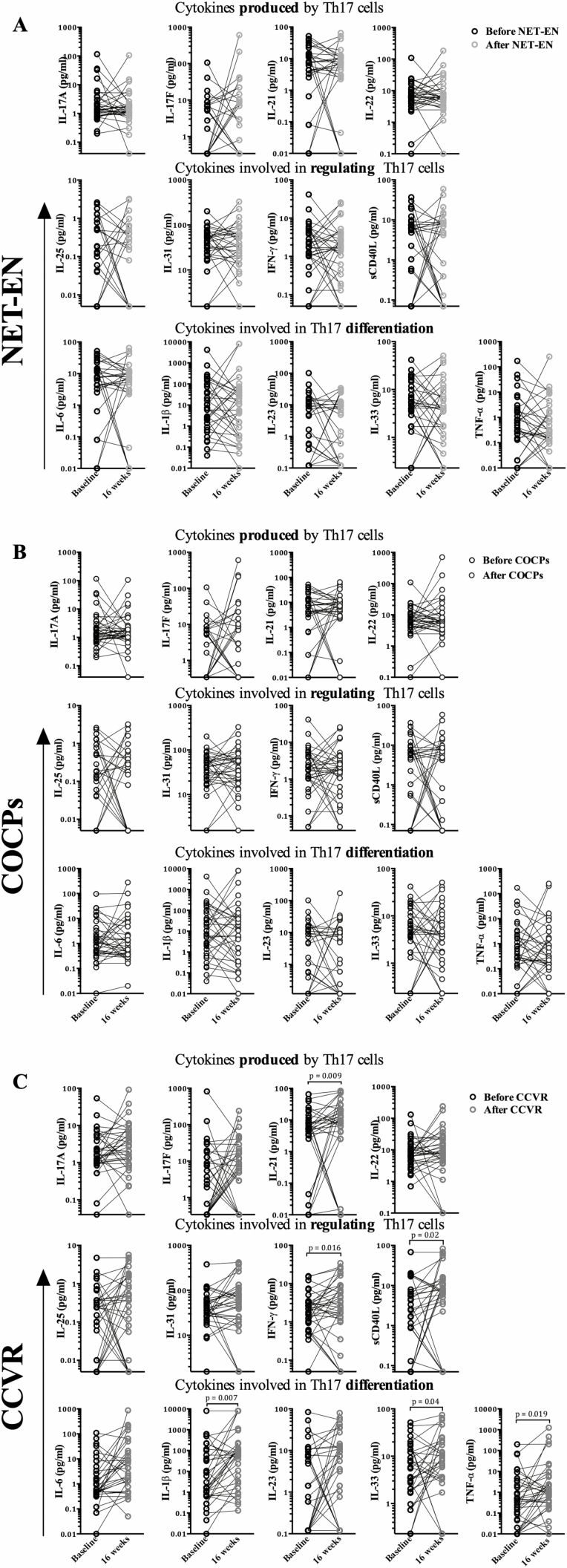
Comparison of genital concentrations of Th17-related cytokines at baseline and crossover (16 weeks). *A*, Concentrations of Th17-related cytokines before and after using NET-EN for 16 weeks. *B*, Concentrations of Th17-related cytokines before and after using COCPs for 16 weeks. *C*, Concentrations of Th17-related cytokines before and after using CCVR for 16 weeks. Cytokine concentrations were compared using a Mann-Whitney U test, and only significant *P* values are displayed (*P* ≤ .05). Cytokines highlighted by the gray-shaded box remained significant after adjusting for multiple comparisons. Abbreviations: CCVR, combined contraceptive vaginal ring; COCPs, combined oral contraceptive pills; IFN-γ, interferon-gamma; IL, interleukin; NET-EN, injectable norethisterone oenanthate; sCD40L, soluble CD40 ligand; Th17, T-helper 17; TNF-α, tumor necrosis factor-alpha.

**Figure 4.**
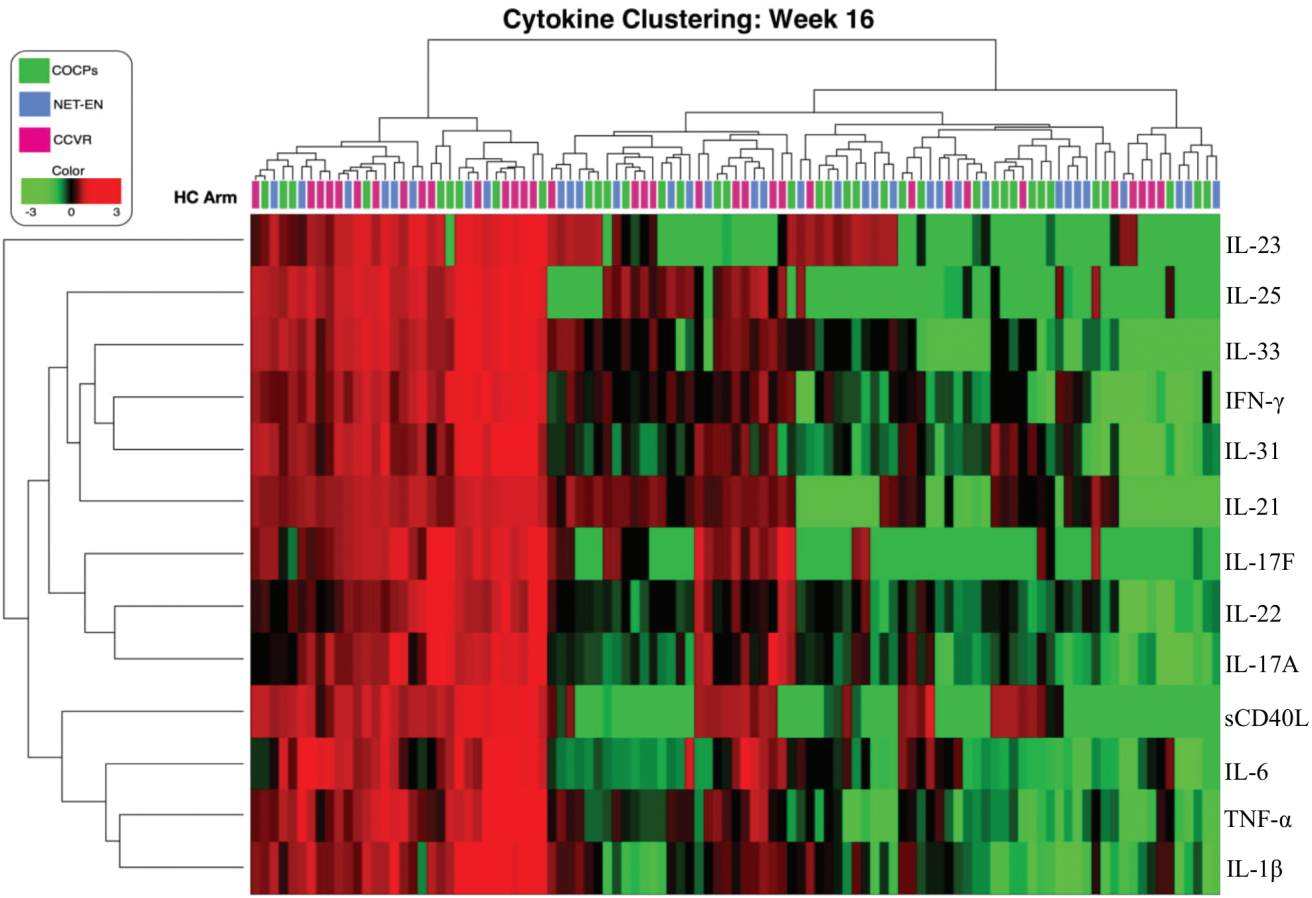
Unsupervised hierarchical clustering of cervicovaginal cytokine concentrations in adolescents randomized to use NET-EN (n = 34; blue blocks), COCPs (n = 37; green blocks), or CCVR (n = 34; pink blocks) for 16 weeks. Adolescents using the CCVR (pink blocks) were enriched in the upregulated cervicovaginal cytokine cluster, compared to those using either NET-EN (blue blocks) or COCPs (green blocks). Cytokine concentrations are indicated using a color scale that ranges from green (low) through black to red (high). The dendrogram above the heat map illustrates the degrees of relatedness between genital cytokine profiles evident within the various adolescents. The dendrogram on the left-hand side of the heat map indicates relationships between the expression profiles of the analyzed cytokines across all of the women assessed in this study. Abbreviations: CCVR, combined contraceptive vaginal ring; COCPs, combined oral contraceptive pills; HC, hormonal contraceptive; IFN-γ, interferon-gamma; IL, interleukin; NET-EN, injectable norethisterone oenanthate; sCD40L, soluble CD40 ligand; TNF-α, tumor necrosis factor-alpha.

In a matched pre-post exposure intent-to-treat analysis, Th17-related cytokine concentrations did not appear to be influenced by NET-EN or COCP use (Figure 3). In contrast, adolescents randomized to CCVR had significantly increased concentrations of inflammatory cytokines (including IL-1β [*P* = .007], TNF-α [*P* = .019], IL-21 [*P* = .009], and IFN-γ [*P* = .016]). In a cross-sectional analysis at 16 weeks, adolescents using NET-EN had similar cytokine concentrations as COCP users (Figure 4). In contrast, adolescents randomized to CCVR had higher concentrations of cytokines, compared to those using NET-EN or COCPs (Figure 4), with significantly higher IL-21 (*P* = .039), IL-17A (*P* = .009), IL-17F (*P* = .033), and IL-33 concentrations (*P* = .03), compared to NET-EN users, and significantly higher concentrations of IL-1β (*P* = .032), TNF-α (*P* = .045), IL-21 (*P* = .049), and IL-6 (*P* = .030), compared to COCP users (Supplementary Table 5). No cytokines correlated with FSH or LH, and only IL-6 was weakly, negatively associated with E2 (Rho = −0.26), but not after adjusting for multiple comparisons (data not shown).

### Longitudinal Effects of Combined Contraceptive Vaginal Rings on T-helper 17 Cells and Cytokines

**Figure 5.**
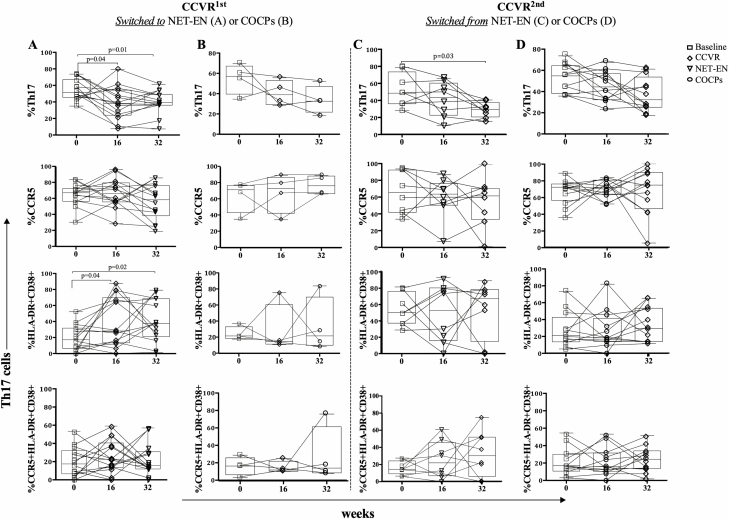
Longitudinal changes in phenotype and activation of cervical Th17 cells in adolescents randomized to use CCVR first and then either crossing to (*A*) NET-EN (n = 14) or (*B*) COCPs (n = 4), and those randomized to use (*C*) NET-EN (n = 8) or (*D*) COCPs (n = 12) first before crossing over to CCVR. Each line connects a single participant across all the 3 visits. Box and whiskers show the median, IQR, and range. The Mann-Whitney U test was applied. Abbreviations: CCVR, combined contraceptive vaginal ring; COCPs, combined oral contraceptive pills; IQR, interquartile range; NET-EN, injectable norethisterone oenanthate; Th17, T-helper 17.

We next assessed whether alterations in Th17 cell frequencies and activation associated with CCVR use resolved after the 16-week shift to another HC (Figures 5A and B). While NET-EN was not associated with changes in Th17 cell frequencies in the first 16 weeks (Figure 5C), switching to NET-EN after using CCVR did not improve Th17 frequencies, which were significantly lower than baseline (*P* = .01; Figure 5A). Moreover, using CCVR first was associated with increased Th17 cell activation, which did not improve after participants changed to NET-EN (comparing HLA-DR+CD38+ at baseline to 16 weeks [CCVR]: *P* = .04; comparing HLA-DR+CD38+ at baseline to 32 weeks [changing from CCVR at 16 weeks to NET-EN]: *P* = .02; Figure 5A). Only 4/40 initially randomized to the CCVR arm and crossed over to COCPs, limiting our ability to investigate the effects associated with moving from CCVR to COCP. No improvements in Th17 frequencies or lowering of Th17 cells activation was noted (Figure 5B). No changes were observed for those randomized to COCPs who switched to CCVR (Figure 5D).

**Figure 6.**
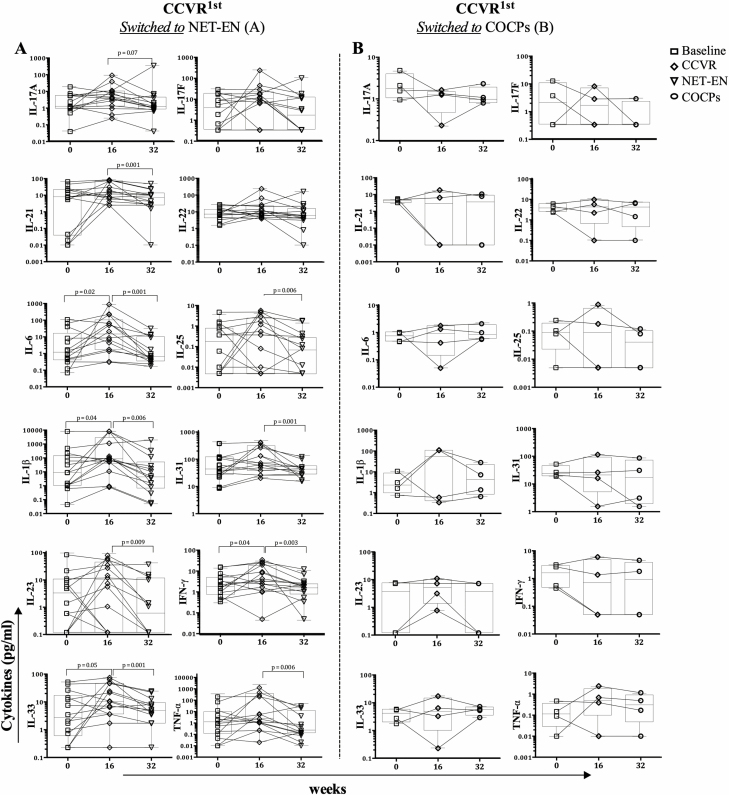
Longitudinal changes in Th17-related cytokine concentrations in adolescents initially randomized to the CCVR and subsequently crossed over to (*A*) NET-EN (n = 21) or (*B*) COCPs (n = 4). The samples were compared using a Mann-Whitney U test. Abbreviations: CCVR, combined contraceptive vaginal ring; COCPs, combined oral contraceptive pills; IFN-γ, interferon-gamma; IL, interleukin; NET-EN, injectable norethisterone oenanthate; Th17, T-helper 17; TNF-α, tumor necrosis factor-alpha.

Next, we investigated whether a shift from CCVR to either NET-EN or COCPs influenced Th17-related cytokines (Figure 6). In this matched analysis, adolescents using CCVR first had higher IL-6, IL-1β, and IL-33 concentrations, compared to baseline (*P* = .02 [adjusted *P* = .02], *P* = .04 [adjusted *P* = .04], *P* = .05 [adjusted *P* = .05], respectively; Figure 6A), which decreased when they switched to NET-EN. Genital IFN-γ concentrations also increased after CCVR use (*P* = .04; adjusted *P* = .04), and decreased after switching to NET-EN (*P* = .003; adjusted *P* = .008). Similarly, IL-21 (*P* = .001; adjusted *P* = .003), IL-23 (*P* = .009; adjusted *P* = .01), IL-25 (*P* = .006; adjusted *P* = .01), IL-31 (*P* = .001; adjusted *P* = .01), and TNF-α (*P* = .006; adjusted *P* = .01) were significantly decreased when adolescents switched from CCVR to NET-EN. Adolescents who initially used CCVR and switched to COCPs had no significant changes in genital cytokine concentrations (Figure 6B), although the numbers were small (n = 4 matched).

**Figure 7.**
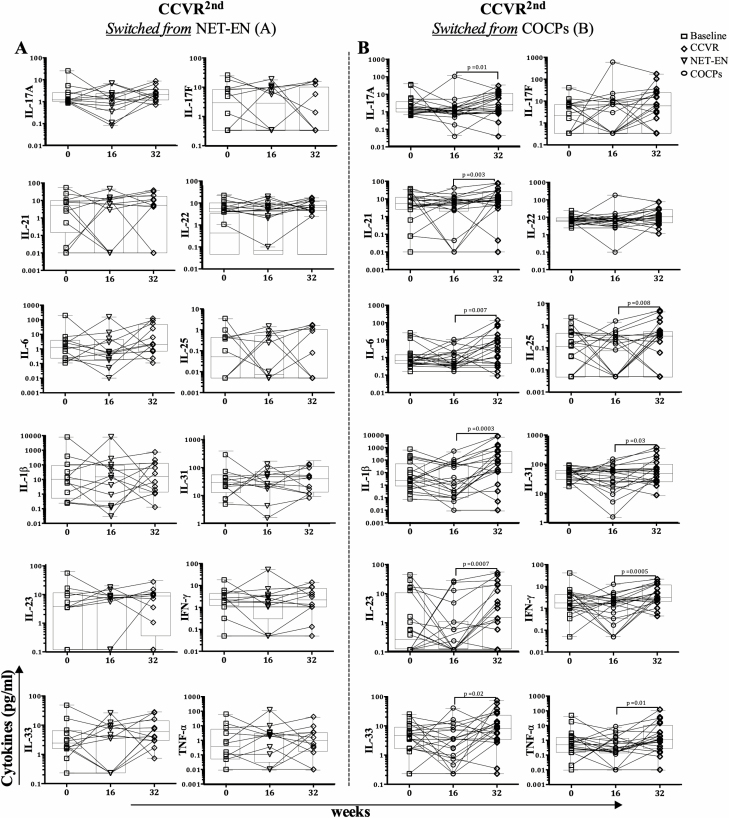
Longitudinal changes in Th17-related cytokine concentrations in adolescents initially randomized to (*A*) NET-EN (n = 24) or (*B*) COCPs (n = 20) and subsequently crossed over to CCVR. The samples were compared using a Mann-Whitney U test. Abbreviations: CCVR, combined contraceptive vaginal ring; COCPs, combined oral contraceptive pills; IFN-γ, interferon-gamma; IL, interleukin; NET-EN, injectable norethisterone oenanthate; Th17, T-helper 17; TNF-α, tumor necrosis factor-alpha.

For adolescents who initially used NET-EN and switched to CCVR, no significant changes in Th17-related cytokine concentrations were observed (Figure 7A). In contrast, significant increases in IL-17A (*P* = .01; adjusted *P* = .01), IL-21 (*P* = .003; adjusted *P* = .007), IL-6 (*P* = .007; adjusted *P* = .01), IL-25 (*P* = .008; adjusted *P* = .01), IL-1β (*P* = .0003; adjusted *P* = .002), IL-31 (*P* = .03; adjusted *P* = .03), IL-23 (*P* = .0007; adjusted *P* = .002), IFN-γ (*P* = .0005; adjusted *P* = .002), IL-33 (*P* = .02; adjusted *P* = .02), and TNF-α (*P* = .01; adjusted *P* = .01) were observed in those adolescents randomized to COCPs and then switched to CCVR (Figure 7B).

## DISCUSSION

Identifying safer HC options is currently a priority, particularly in adolescent and young women, as they are at high risk of both HIV and unintended pregnancies. We examined the effects of HCs on cervical Th17 cell frequencies, activation, and Th17-related cytokines from adolescents, since Th17 cells have been identified as being a highly HIV-susceptible CD4+ T-cell subtype [15, 16]. To our knowledge, this is the first clinical study to characterize CCR6+ CCR10- Th17 cells in adolescents. Th17 cells from the cervixes of adolescents were a major mucosal CD4+ T-cell subset, with high frequencies expressing CCR5, as well as CCR5 together with the activation marker CD38, compared to CCR6- CCR10- CD4+ T cells. Although long-acting progestin-only injectable HCs have been associated with increased HIV risks in several large meta-analyses [2, 20], we found that NET-EN use for 16 weeks did not influence the frequency of Th17 cells or CCR5 expression on these cells, and did not result in changes in Th17-related cytokines as compared to matched baseline, similar to COCPs. In contrast, the use of CCVRs was associated with significant increases in both the activation status of CCR5+ Th17 cells and increased concentrations of several inflammatory and Th17-related cytokines, including IL-1β and IL-21, compared to matched baseline measures, and in a cross-sectional comparison between HC groups at crossover. Further, we found that CCVR-associated inductions in cytokines were significantly reduced in adolescents when they subsequently switched to NET-EN, confirming that the removal of the ring resolved this response. The transition to CCVR at crossover resulted in significant increases in several cytokines in women who did not have elevated inflammation previously.

Among the many functions of Th17-related cytokines is to recruit Th17 cells to mucosal surfaces, including the genital tract [21]. Amongst the cytokines produced by Th17 cells (IL-17A, IL-17F, IL-21, and IL-22), IL-17F was less affected by the use of CCVR. Although both IL-17A and IL-17F play critical roles in tissue inflammation by inducing the release of proinflammatory cytokines, IL-17F has been reported to be less potent at inducing cytokines [22–24]. The increase in Th17-related cytokines observed when participants initiated CCVR might suggest both hormonal and mechanical responses to the copolymer evatane vaginal ring (forming the CCVR used here) in the vagina. Although neutrophil infiltration was not measured directly, Th17 cells recruit neutrophils by producing IL-17A and IL-17F [25]. The activation of Th17 cells has been reported to result in a large amount of inflammatory cytokine production, and increased frequencies of highly activated cervical Th17 cells may partially explain why CCVR use was associated with increased genital Th17-related cytokine concentrations. CCVR use has previously been reported to increase vaginal wetness, and some have suggested that this is due to inflammation [26, 27]. Several studies have assessed whether vaginal rings delivering antiretrovirals induce physiological or inflammatory changes, although mucosal immune cells were not measured in these studies [28–30]. In macaques, insertion of a silicone intravaginal ring was not associated with changes in IL-6 and IL-8 concentrations, although biofilm development was noted [31].

With elevated cytokines during CCVR use, we would expect increased numbers and/or frequencies of Th17 cells or total CD4+ T cells. It was therefore surprising that the overall frequencies of total genital CD4+ and Th17-like T cells were significantly reduced in adolescents assigned to the CCVR arm, although CCVR use was associated with increased proportions of activated and CCR5-expressing Th17 cells. The impact of CCVR on endogenous FSH and LH concentrations may provide an explanation for this, as FSH appeared to be a major, positive predictor of cervical Th17 cell frequencies. FSH plays a role in the relaxation and opening of the cervix following ovulation [32], and it is interesting to speculate that this may also be a time of heightened vulnerability to infection, necessitating higher concentrations of mucosal immune surveillance. CCVR and COCPs have previously been reported to reduced frequencies of CD207+ Langerhans cells in the vagina [33], which are known to activate cutaneous Th17 responses in response to bacterial infections [34]. While the cytokine changes associated with CCVR use may suggest that it is recognized as a foreign object, which may induce microbial changes, lower concentrations of endogenous hormones might also contribute.

This study has several limitations. Because the parent study was designed to assess the acceptability of CCVRs in South African adolescents, they were randomized for the first 16 weeks only, after which adolescents chose their HC option for the remaining 16 weeks. It was not possible to explore different sequences of HCs as only the first phase (16 weeks) was randomized, after which the parent study’s primary outcome was to evaluate factors determining contraceptive choices in adolescents, and participants were asked at 16 weeks about factors influencing their “desire to change contraceptive methods” and “adherence” in a detailed questionnaire. Th17 frequencies and cytokine changes were not the primary outcomes of the study and, as such, the trial was not powered to detect differences. Despite this, some significant and interesting differences in these biomedical outcomes were noted. Only a subset of adolescents was contraceptive-naive at baseline, as participants had to be sexually active to be eligible and most had initiated a HC. For participants <18 years of age, the recruitment process was difficult, as parental consent—and, thus, disclosure of sexual activity—was required. The relatively modest retention rate was a limitation, although this is an intrinsic difficulty in enrolling adolescents. In addition, substantial heterogeneity in cytokine measurements and Th17 frequencies between and within individuals were noted, and it would have been desirable to include multiple time points for each woman per HC method, in order to reduce some of the noise in these assays, even though each woman served as her own control.

In conclusion, adolescents randomized to the CCVR appeared to have significantly elevated inflammatory responses (cytokines and activation), suggestive of an HIV risk, although frequencies of cervical Th17 cells were lower compared to those participants using NET-EN and COCPs, possibly tempering this finding. This emphasizes the need to understand the biomedical impacts of new HC modalities in adolescents, particularly in settings of high risk for HIV infection.

## Supplementary Data

Supplementary materials are available at *Clinical Infectious Diseases* online. Consisting of data provided by the authors to benefit the reader, the posted materials are not copyedited and are the sole responsibility of the authors, so questions or comments should be addressed to the corresponding author.

ciz1063_suppl_Supplementary_Figure_S1

ciz1063_suppl_Supplementary_Figure_S2

ciz1063_suppl_Supplementary_Table_S1

ciz1063_suppl_Supplementary_Table_S2

ciz1063_suppl_Supplementary_Table_S3

ciz1063_suppl_Supplementary_Table_S4

ciz1063_suppl_Supplementary_Table_S5

ciz1063_suppl_Supplementary_Legends

## Notes

***Acknowledgments.*** The authors thank the uCHOOSE study team, particularly Sisters Penelope Ngcobo and Janine Nixon and Drs Keshani Naidoo and Eve Mendel. They thank Wanani Mubilanzila, Thandi Magwai, Valerie Masete, Madeleine Heller, and Rachel Esra for assistance with processing samples. They are grateful to all the adolescent girls who participated in this study.

***Disclaimer.*** The study funders had no role in the study design, data collection, analysis, interpretation, or writing of the manuscript.

***Financial support.*** This work was supported by funding from the US National Institute of Health and SA Medical Research Council (grant number RO1 HD083040 to J.-A. S. P. and H. B. J.). The NuvaRing was donated by Merck for this study.

***Potential conflicts of interest.*** The authors: No reported conflicts of interest. All authors have submitted the ICMJE Form for Disclosure of Potential Conflicts of Interest.
