# Supplementary material for: Impact of Hormonal Contraceptives on Cervical T-helper 17 Phenotype and Function in Adolescents: Results from a Randomized, Crossover Study Comparing Long-acting Injectable Norethisterone Oenanthate (NET-EN), Combined Oral Contraceptive Pills, and Combined Contraceptive Vaginal Rings
Source: Clin Infect Dis. 2019 Nov 2;71(7):e76–87. doi: 10.1093/cid/ciz1063 (PMC7755094; doi:10.1093/cid/ciz1063)
Supplement: ciz1063_suppl_Supplementary_Table_S2 [file ciz1063_suppl_supplementary_table_s2.docx]

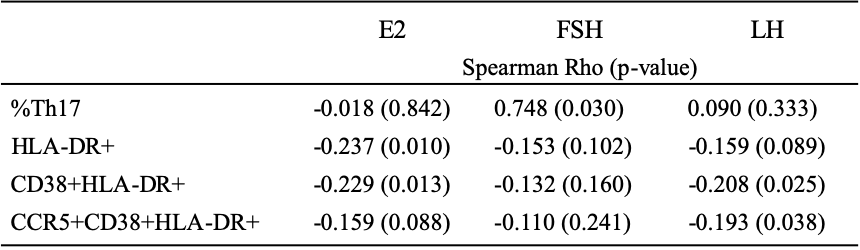


Supplementary Table 2. Endogenous hormone concentrations and cervical Th17 cell characteristics
