# Supplementary material for: Impact of Hormonal Contraceptives on Cervical T-helper 17 Phenotype and Function in Adolescents: Results from a Randomized, Crossover Study Comparing Long-acting Injectable Norethisterone Oenanthate (NET-EN), Combined Oral Contraceptive Pills, and Combined Contraceptive Vaginal Rings
Source: Clin Infect Dis. 2019 Nov 2;71(7):e76–87. doi: 10.1093/cid/ciz1063 (PMC7755094; doi:10.1093/cid/ciz1063)
Supplement: ciz1063_suppl_Supplementary_Table_S4 [file ciz1063_suppl_supplementary_table_s4.docx]

Supplementary Table 4. Endogenous hormone concentrations and STI/BV prevalence, according to study arm

|  | | CCVR  n=36 | | | | NET-EN  n=23 | | | COCPs  n=6 | | P-value | | |
| --- | --- | --- | --- | --- | --- | --- | --- | --- | --- | --- | --- | --- | --- |
| Endogenous Hormones: | |  | | | |  | | |  | |  | | |
| FSH (U/L) | | 1.1 (0.1-3.1) | | | | 5.2 (3.8-6.0) | | | 3.4 (1.6-5.1%) | | <0.0001 | | |
| LH (IU/L) | | 0.5 (0.5-3.3) | | | | 4.8 (2.9-5.4) | | | 4.0 (0.5-7.1) | | <0.0001 | | |
| E2 (pmol/l) | | 48 (37-159) | | | | 103 (66-152) | | | 101 (50-341) | | 0.05 | | |
| STIs/BV: | |  | | | |  | | |  | |  | | |
| *Chlamydia trachomatis* | | 8 (22.9%) | | | | 4 (11.4%) | | | 4 (10.8%) | | | 0.278 | |
| *Neisseria gonorrhoeae* | | | 7 (20.0) | | 1 (2.9%) | | | 2 (5.4%) | | | | | 0.062 |
| *Trichomonas vaginalis* | | | 1 (2.9%) | | 1 (2.9%) | | | 1 (2.7%) | | | | | 1.000 |
| *Mycoplasma genitalium* | | | 0 (0.0%) | | 4 (11.4%) | | | 1 (2.7%) | | | | | 0.081 |
| BV Nugent 7-10 | | | 15 (42.9%) | | 16 (45.7%) | | | 12 (32.4%) | | | | | 0.697 |
|  |  | | |  | | |  | | |  | | | |
