# Supplementary material for: Impact of Hormonal Contraceptives on Cervical T-helper 17 Phenotype and Function in Adolescents: Results from a Randomized, Crossover Study Comparing Long-acting Injectable Norethisterone Oenanthate (NET-EN), Combined Oral Contraceptive Pills, and Combined Contraceptive Vaginal Rings
Source: Clin Infect Dis. 2019 Nov 2;71(7):e76–87. doi: 10.1093/cid/ciz1063 (PMC7755094; doi:10.1093/cid/ciz1063)
Supplement: ciz1063_suppl_Supplementary_Table_S5 [file ciz1063_suppl_supplementary_table_s5.docx]

Supplementary Table 5. Cytokine concentrations after 16-weeks of using NET-EN, COCPs or CCVR

| Cytokines and function | NET-EN^#^  Median (IQR) | COCPs  Median (IQR) | P-value | CCVR  Median (IQR) | P-value |
| --- | --- | --- | --- | --- | --- |
|  | | | | | |
| Cytokines produced by Th17 cells | | | | | |
| IL-17A | 1.05 (0.62 - 2.50) | 1.48 (1.01 - 2.24) | 0.225 | 3.24 (0.91 - 7.69)* | 0.009 |
| IL-17F | 0.34 (0.34 - 10.80) | 0.80 (0.34 - 10.14) | 0.658 | 6.83 (0.34 - 23.86)* | 0.033 |
| IL-21 | 4.51 (0.01 - 16.10) | 7.22 (2.35 - 11.43) | 0.519 | 12.12 (3.75 - 23.59)* | 0.039 |
| IL-22 | 7.00 (4.05 -10.81) | 6.43 (4.32 - 11.73) | 0.852 | 9.22 (4.57 - 21.83) | 0.173 |
|  |  |  |  |  |  |
| Cytokines involved in differentiation of Th17 cells | | | | | |
| IL-6 | 1.01 (0.17 - 13.56) | 1.01 (0.36 - 6.51) | 0.943 | 3.43 (0.69 - 28.10) | 0.076 |
| IL-1β | 10.68 (0.25 - 76.34) | 11.32 (0.36 - 56.51) | 0.970 | 61.12 (2.70 - 152.0) | 0.061 |
| IL-23 | 3.99 (0.12 - 11.43) | 0.59 (0.12-9.54) | 0.735 | 4.56 (0.12 - 13.27) | 0.387 |
| IL-33 | 3.50 (0.23 - 10.13) | 3.65 (1.08 - 9.84) | 0.605 | 8.51 (3.43 - 22.19)* | 0.034 |
| TNF-α | 1.38 (0.03 - 3.62) | 0.32 (0.13 - 2.41) | 0.583 | 1.21 (0.29 - 6.69) | 0.344 |
|  | | | | | |
| Cytokines involved in regulating Th17 cells | | | | | |
| IL-25 | 0.01 (0.01 - 0.62) | 0.01 (0.01 - 0.45) | 0.817 | 0.37 (0.01 - 1.51) | 0.059 |
| IL-31 | 26.63 (17.14 - 61.97) | 34.99 (21.03 - 65.77) | 0.373 | 54.09 (19.30 - 108.10) | 0.131 |
| IFN-γ | 2.25 (0.51 - 3.99) | 1.81 (0.84 - 4.03) | 0.947 | 2.95 (1.21 - 8.73) | 0.226 |
| sCD40L | 0.07 (0.07 - 8.90) | 0.32 (0.13 - 2.41) | 0.447 | 6.70 (0.07-11.93) | 0.121 |

^#^NET-EN was considered the comparator group for statistical comparisons with COCPs and CCVR;

*indicate comparisons with NET-EN that were significant before adjusting for multiple comparisons.
