# Supplementary material for: Impact of Hormonal Contraceptives on Cervical T-helper 17 Phenotype and Function in Adolescents: Results from a Randomized, Crossover Study Comparing Long-acting Injectable Norethisterone Oenanthate (NET-EN), Combined Oral Contraceptive Pills, and Combined Contraceptive Vaginal Rings
Source: Clin Infect Dis. 2019 Nov 2;71(7):e76–87. doi: 10.1093/cid/ciz1063 (PMC7755094; doi:10.1093/cid/ciz1063)
Supplement: ciz1063_suppl_Supplementary_Legends [file ciz1063_suppl_supplementary_legends.docx]

Supplementary Figure 1. Gating strategy for phenotyping cervical cytobrush-derived T cells. Gates were drawn based on fluorescence-minus-one (FMO) controls. A time gate was set followed by a singlet gate to exclude doublets. Next, live CD3 cells were gated on followed by CD4+ and CD8+ T cell gates. Chemokine receptors CCR6 and CCR10 were stained to identify different CD4+ T cell subsets including Th17 cells. Activated cells were defined as those expressing CD38 or/and HLA-DR. The CCR5 chemokine receptor needed for HIV-1 entry was also stained.

Supplementary Figure 2. Phenotype of CD4+ T cells before (baseline) and after (16 weeks) being on (A) NET-EN (B) COCPs and (C) CCVR. The frequencies of CD4+ T cells and expression of CCR5, CD38 and HLA-DR on these cells were assessed before and after 16 weeks of adolescents being on the randomized HC. A Wilcoxon matched-pairs signed rank test was applied and a p-value ≤0.05 was considered significant.
